# Second Malignant Neoplasms in Patients With Rhabdomyosarcoma

**DOI:** 10.3389/fonc.2021.757095

**Published:** 2021-10-14

**Authors:** Hongnan Zhen, Zhikai Liu, Hui Guan, Jiabin Ma, Wenhui Wang, Jing Shen, Zheng Miao, Fuquan Zhang

**Affiliations:** Department of Radiation Oncology, Peking Union Medical College Hospital, Chinese Academy of Medical Sciences & Peking Union Medical College, Beijing, China

**Keywords:** survival analysis, chemotherapy, radiation therapy, second malignant neoplasm, rhabdomyosarcoma

## Abstract

**Objective:**

Rhabdomyosarcoma (RMS) is a rare malignant tumor. The main treatment modality is comprehensive with chemotherapy, radiotherapy, and surgery. With the advancement in recent decades, patient survival has been prolonged, and long-term complications are attracting increasing attention among both physicians and patients. This study aimed to present the survival of patients with RMS and analyze the risk factors for the development of a second malignant neoplasm (SMN).

**Methods:**

The Surveillance, Epidemiology, and End Results (SEER) Program 18 registry database from 1973 to 2015 of the National Cancer Institute of the United States was used for the survival analyses, and the SEER 9 for the SMN analysis.

**Results:**

The 5-, 10-, and 20-year overall survival rates of the patients with RMS were 45%, 43%, and 33%, respectively. The risk of SMN was significantly higher in patients with RMS compared to the general population (SIR=1.95, 95% CI: 1.44 – 2.57, *p* < 0.001). The risk of developing SMN was increased in multiple locations, including the bones and joints (SIR = 35.25) soft tissues including the heart (SIR = 22.5), breasts (SIR = 2.10), male genital organs (SIR = 118.14), urinary system (SIR = 2.36), brain (SIR = 9.21), and all nervous system organs (SIR = 8.59). The multivariate analysis indicated that RMS in the limbs and earlier diagnosis time were independent risk factors for the development of SMN. Patients with head and neck (OR = 0.546, 95% CI: 0.313 – 0.952, *p* = 0.033) and trunk RMS (OR = 0.322, 95% CI: 0.184 – 0.564. *p* < 0.001) and a later diagnosis time were less likely to develop SMN (OR = 0.496, 95% CI: 0.421 – 0.585, *p* < 0.001).

**Conclusion:**

This study describes the risk factors associated with the development of SMN in patients with RMS, which is helpful for the personalized screening of high-risk patients with RMS.

## Introduction

Rhabdomyosarcoma (RMS), although rare, is a common childhood cancer and is the most common soft tissue sarcoma among children. The overall incidence among those aged < 20 years is 4.5 cases per million people (1). In the United States, approximately 350 new cases are recorded each year. According to the data of the Surveillance Epidemiology and End Results (SEER) program, the incidence of RMS varies depending on age and histopathology (2).

Significant advances have been made over the past four decades in the treatment of RMS. The majority of children with the early-stage disease will essentially be cured. Because of the collaborative effort dedicated to clinical trials, the current 3-year overall survival rate of patients with high-risk RMS is approximately 80% and treatment failure is largely attributed to local relapse. Therefore, increasing the local control rate remains challenging in the treatment of RMS (1).

Increasing the dosage of cytotoxic drugs may lead to prognostic improvement but is accompanied by increased side effects, including long-term ones such as Second malignant neoplasm (SMN), which has particularly drawn the attention of both physicians and RMS survivors (1). There is a need to assess the risk of SMN in patients with RMS. However, it is difficult to do so in a single-center cohort with a long-term follow-up due to the limited incidence. Therefore, this study aimed to assess the risk of SMN development in patients with RMS through this population-based study using clinical data from the SEER database.

## Material And Methods

Data of patients with RMS, including treatment information between 1973 and 2015 was collected from the SEER 18 database using the SEER*Stat software (version 8.3.6). The inclusion criteria were patients with all types of RMS (pleomorphic, mixed-type, embryonal, spindle cell, alveolar, with ganglionic differentiation, and Not otherwise specified (NOS)) according to the following codes: ICD-O-3Hist/behave=“8900/3:rhabdomyosarcoma, NOS”,”8901/3:pleomorphic rhabdomyosarcoma, adult-type”, “8902/3:mixed type rhabdomyosarcoma”, “8910/3:Embryonal rhabdomyosarcoma,NOS”, “8912/3:spindle cell rhabdomyosarcoma”, “8920/3:alveolar rhabdomyosarcoma”, “8921/3:rhabdomyosarcoma with ganglionic differentiation”. Moreover, patients with clear histologic subtypes and classifications, as well as those with complete follow-up information were included. The exclusion criteria were cases diagnosed by autopsy and those with incomplete follow-up information.

The survival time used in this study was overall survival (OS). SMNs were defined as other malignant tumors that were present at least 2 months after the diagnosis of RMS. Information at diagnosis, including age, sex, race, histologic subtypes and differentiation level, year of diagnosis, major location, radiotherapy, chemotherapy, survival time, and survival condition was collected. Authorization was obtained from the SEER website to collect the data from the database and no additional ethical approval was required.

### Statistical Analysis

Categorical data was presented as frequencies and percentages. The Kaplan-Meier method was used for survival analysis. Univariate analyses were performed using the log-rank test, and multivariate using the Cox regression model. Independent risk factors for the development of SMNs were determined using logistic regression analysis. The standardized incidence ratio (SIR) was calculated using SEER*Stat and was analyzed and presented with 95% confidence interval CI. All statistical analyses were performed using SPSS 22.0 (SPSS, Inc., Chicago, IL). A *p-value* of ≤ 0.05 was used to indicate statistical significance.

## Results

### Demographic and Clinical Characteristics of Study Patients

A total of 4,787 pathologically diagnosed patients with RMS were included in this study. The demographic and clinical characteristics of all patients with RMS and those with SMNs are shown in **Table 1**. The trunk was the most common primary site (50.6%, n = 2,420), followed by the head and neck (36.1%, n = 1,728) and the limbs (12.2%, n = 586). In terms of histologic subtypes, embryonal was the most common (41.0%, n = 1,964), followed by NOS (36.5%, n = 1,746) and alveolar type (22.5%, n = 1,077). The risk of primary SMNs was 34.1% in the head and neck and 32.9% in both the limbs and the trunk. Patients with SMN were most likely to have NOS (54.7%) and embryonal (36.0%) as opposed to alveolar type.

**Table 1:** Demographic and clinical characteristics of patients with rhabdomyosarcoma.

Variables	Categories	Cohort (%)	
		All RMS patients (n = 4,787)	SMN (n = 86)
Sex	Male	2,678 (55.9)	48 (55.8)
	Female	2,109 (44.1)	38 (44.2)
Age at RMS diagnosis (years)	0 – 19	2,646 (55.3)	39 (45.3)
	20 – 39	639 (13.3)	11 (12.8)
	40 – 59	568 (11.9)	15 (17.4)
	≥60	934 (19.5)	21 (24.4)
Year of diagnosis	1973 – 1979	435 (9.1)	30 (34.9)
	1980 – 1989	479 (10.0)	17 (19.8)
	1990 – 1999	686 (14.3)	22 (25.6)
	2000 – 2009	1,926 (40.2)	12 (14.0)
	2010 – 2015	1,261 (26.3)	5 (5.8)
Primary site	Head and Neck	1,728 (36.1)	29 (34.1)
	Trunk	2,420 (50.6)	28 (32.9)
	Limbs	586 (12.2)	28 (32.9)
Histologic subtypes	NOS	1,746 (36.5)	47 (54.7)
	Embryonal	1,964 (41.0)	31 (36.0)
	Alveolar	1,077 (22.5)	8 (9.3)
Race	White	3,694 (77.2)	67 (77.9)
	Black	715 (14.9)	11 (12.8)
	Others	353 (7.4)	8 (9.3)
Radiotherapy	Yes	2,556 (53.4)	48 (55.8)
	No/Unknown	2,231 (46.6)	38 (44.2)
Chemotherapy	Yes	3,633 (75.9)	54 (62.8)
	No/Unknown	1,154 (24.1)	32 (37.2)

SEER 18, Surveillance, Epidemiology, and End Results Program 18; RMS, rhabdomyosarcoma; SMN, second malignant neoplasm; NOS, not otherwise specified.

### OS of Patients With RMS

**Figure 1:**
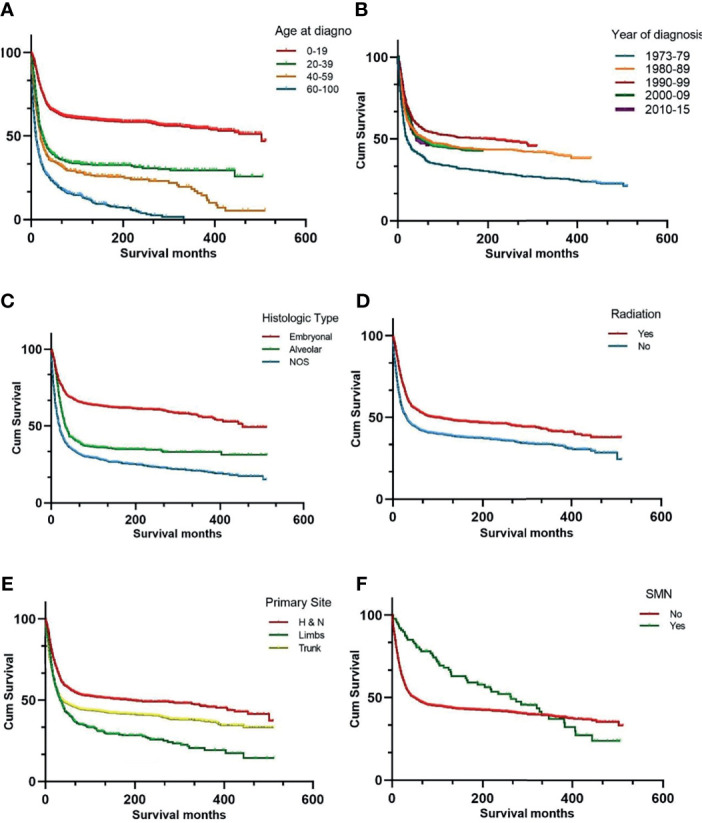
Kaplan-Meier curves of overall survival stratified by age at diagnosis **(A)** year of diagnosis **(B)** histologic subtypes **(C)** radiation therapy **(D)** primary site **(E)** and SMN **(F)**. SMN, second malignant neoplasm; NOS, not otherwise specified.

The 5-, 10-, and 20-year OS rates for the entire RMS cohort were 45%, 43%, and 33%, respectively. The survival data of the different variables are presented in **Figure 1**. Univariate analysis of the independent risk factors for mortality was initially conducted, and those with *p* < 0.1 were included in the multivariate analysis. The statistically significant predictors of survival were sex (*p* = 0.007), age (*p* < 0.001) and year of diagnosis (*p* < 0.001), primary site (*p* < 0.001), RMS type (*p* < 0.001), radiotherapy (*p* < 0.001), chemotherapy (*p* < 0.001), and degree of differentiation (*p* < 0.001), and SMN (*p* = 0.013). After adjusting for other factors, the multivariate analysis indicated that SMN (hazard ratio (HR) = 2.43, 95% CI: 1.369 – 4.312, *p* = 0.002) was independently associated with decreased survival.

### Risk of SMN in Patients With RMS

Overall, 86 patients developed SMNs with an SIR of 1.95 (95% CI: 1.44 – 2.57, *p* < 0.001) and the SIR of solid tumors was 1.95 (95% CI: 1.54 – 2.44 and *p* < 0.05). Bones and joints (SIR = 35.25, 95% CI: 14.17 – 72.63, *p* < 0.001), soft tissues including the heart (SIR = 22.5, 95% CI: 10.29 – 42.7, *p* < 0.05), breasts (SIR = 2.10, 95% CI: 1.05 – 3.75, *p* < 0.05), male genital organs (SIR = 118.14, 95% CI: 14.31 – 426.78, *p* < 0.05), the urinary system (SIR = 2.36, 95% CI: 1.02 – 4.66, *p* < 0.05), the brain (SIR = 9.21, 95% CI: 3.98 – 18.16, *p* < 0.05), and all nervous system organs (SIR = 8.59, 95% CI: 3.71 – 16.93, *p* < 0.05) all had an increased risk of SMN. The most common hematological SMNs were non-lymphocytic leukemia (SIR = 5.24, 95% CI: 1.43 – 13.42, *p* < 0.05), and myeloid and monocytic leukemia (SIR = 5.90, 95% CI: 1.61 – 15.1, *p* < 0.05).

### Analysis of SMN Risk Factors

The univariate analysis showed that the year of diagnosis (*p* < 0.001), primary site (*p* < 0.001), and chemotherapy (*p* = 0.006) were associated with SMN, whereas radiotherapy (*p* = 0.58) and histologic type (*p* = 0.30) were not. The multivariate analysis indicated that RMS in the limbs and earlier diagnosis time were independent risk factors for the development of SMN. Patients with head and neck (SIR = 0.546, 95% CI: 0.313 – 0.952, *p* = 0.033), trunk RMS (SIR = 0.322, 95% CI: 0.184 – 0.564, *p* < 0.001), and a later diagnosis time were less likely to develop SMN (SIR = 0.496, 95% CI: 0.421 – 0.585, *p* < 0.001). The demographic and clinical characteristics of SMNs with RMS by years are shown in **Table 2**.

**Table 2:** Demographic and clinical characteristics of SMNs with RMS by years.

Variables	Categories	Year of diagnosis					
		1973 – 1979	1980 – 1989	1990 – 1999	2000 – 2009	2010 – 2015	Total(n = 86)
Sex	Male	17 (19.8)	8 (9.3)	14 (16.3)	5 (5.8)	4 (4.7)	48 (55.8)
	Female	13 (15.1)	9 (10.5)	8 (9.3)	7 (8.1)	1 (1.2)	38 (44.2)
Age at RMS diagnosis (years)	0 – 19	11 (12.8)	8 (9.3)	11 (12.8)	6 (7.0)	3 (3.5)	39 (45.3)
	20 – 39	3 (3.5)	2 (2.3)	4 (4.7)	1 (1.2)	1 (1.2)	11 (12.8)
	40 – 59	5 (5.8)	3 (3.5)	2 (2.3)	4 (4.7)	1 (1.2)	15 (17.4)
	≥ 60	11 (12.8)	4 (4.7)	5 (5.8)	1 (1.2)	0 (0)	21 (24.4)
Primary site	Head and Neck	12 (14.0)	5 (5.8)	6 (7.0)	5 (5.8)	1 (1.2)	29 (34.1)
	Trunk	4 (4.7)	7 (8.1)	10 (11.6)	4 (4.7)	3 (3.5)	28 (32.9)
	Limbs	13 (15.1)	5 (5.8)	6 (7.0)	3 (3.5)	1 (1.2)	28 (32.9)
Histologic subtypes	NOS	17 (20)	9 (10.5)	15 (17.4)	3 (3.5)	3 (3.5)	47 (54.7)
	Embryonal	11 (12.8)	7 (8.1)	6 (7.0)	6 (7.0)	1 (1.2)	31 (36.0)
	Alveolar	2 (2.3)	1 (1.2)	1 (1.2)	3 (3.5)	1 (1.2)	8 (9.3)
Race	White	25 (29.1)	15 (17.4)	14 (16.3)	9 (10.5)	4 (4.7)	67 (77.9)
	Black	4 (4.7)	1 (1.2)	4 (4.7)	1 (1.2)	1 (1.2)	11 (12.8)
	Other	1 (1.2)	1 (1.2)	4 (4.7)	2 (2.4)	0 (0)	8 (9.3)
Radiation therapy	Yes	16 (18.6)	12 (14.0)	13 (15.1)	5 (5.8)	2 (2.3)	48 (55.8)
	No/Unknown	14 (16.3)	5 (5.8)	9 (10.5)	7 (8.1)	3 (3.5)	38 (44.2)
Chemotherapy	Yes	14 (16.3)	9 (10.5)	15 (17.4)	11 (12.8)	5 (5.8)	54 (62.8)
	No/Unknown	16 (18.6)	8 (9.3)	7 (8.1)	1 (1.2)	0 (0)	32 (37.2)

RMS, rhabdomyosarcoma; SMN, second malignant neoplasm; NOS, not otherwise specified.

## Discussion

In this study, we found that the development of SMNs, primary site, histologic subtypes, age, radiotherapy, and the degree of differentiation were independent risk factors affecting the prognosis of patients with RMS. Compared to the general population (18 states in the United States participating in SEER), patients with RMS had a significantly increased risk of developing SMNs, and risk factors found to be associated with their development included primary site and the time of diagnosis.

In recent decades, survival has significantly improved among patients with RMS. This has been mainly due to the multidisciplinary treatment approach, which includes optimal cytotoxic drug combinations, and advances in radiotherapy. The greater number of RMS survivors leads to an increased risk of late complications, such as SMNs. The results of our study suggested that the year of diagnosis was a risk factor affecting the incidence of SMN, and the incidence of SMN was increased with the prolongation of survival time. Heyn et al. found that the incidence rate of SMN increased significantly with increased time since diagnosis (3). Studies conducted on childhood tumors have also reached similar conclusions of the prolonged time of several years, and sometimes even decades of follow-up to observe the occurrence of SMN. These studies showed that the 10-year cumulative incidence rate of SMN in patients with childhood tumors was approximately 1.2%–1.7%, the 20-year cumulative incidence rate was 3.3%–4.3%, and the 25-year cumulative incidence rate was 3.7%–12.1% (3–10). The above studies indicate that the incidence of SMN will gradually increase as the survival period of childhood tumor survivors increases. We believe that there are two possible reasons. One is that the SMN (such as leukemia, radiation carcinogenesis, etc.) caused by therapeutic factors usually takes years to decades to manifest. The other is that survivors are getting older, and the incidence of SMN is increased due to non-therapeutic factors represented by heredity. The above two factors work together to cause the diagnosis time to become a factor that affects the occurrence of SMN. Moreover, we found that patients with RMS of the limbs were more likely to develop SMNs. There have not been relevant research studies that verify this conclusion, and the effects of different primary sites of RMS on SMN have not been explored. Because RMS of the extremities is correlated with a higher rate of treatment failure, we suppose that the higher risk of SMN of the extremities could be related to the cumulative dose of cytotoxic drugs and the intensity of radiotherapy.

The results of our study showed that radiotherapy and chemotherapy were not the independent risk factors for the development of SMNs. Other studies also have the same results (5). In addition, for most types of radiotherapy, the SMN sites were located outside the field of irradiation. Therefore, it was speculated that radiotherapy did not directly lead to an increase in the incidence of SMN (5). But the mainstream view is that radiotherapy is a risk factor for SMN (11–14), they found that the radiotherapy doses may be correlated with the risk of SMN. Moreover, radiation is carcinogenic but the mechanism is unclear, so it is difficult to exclude the influence of it on SMN. Whether chemotherapy affects independent risk factors for SMN is also controversial, and mainstream views also believe that chemotherapy can cause SMN. Cytotoxic drugs, such as cyclophosphamide and etoposide, have been reported to significantly increase the risk of secondary malignant leukemia in patients with RMS (15). The incidence of SMNs was even lower in patients who used actinomycin for treatment, suggesting that this drug may have some protective effects (15). Our results showed that chemotherapy was a risk factor for SMN in a univariate analysis (p=0.006), but it was not statistically significant in multivariate analysis (p>0.05).

Consistent with Archer et al. (16), our study found that the histologic type was not a risk factor of SMN (*p*=0.3). He found a significant increase in the incidence of SMN in young children (<10 years) with embryonal histology tumors receiving radiotherapy, further supporting the hypothesis of the potential existence of a subgroup of children with constitutional risk. However, due to the small number of SMN patients, no further verification can be performed. One possible reason is that patients with embryonal RMS are prone to cancer syndromes, such as Li-Fraumeni, which is a correlation that many studies have confirmed (6, 8–10). Li-Fraumeni syndrome is a hereditary autosomal dominant disease that has been closely associated with RMS and with mutations in the tumor suppressor *TP53* gene. Common SMNs in patients with RMS include breast cancer, osteosarcoma, brain tumors, leukemia, and adrenocortical malignancy, which are very similar to the clinical characteristics of Li-Fraumeni syndrome. Li-Fraumeni syndrome is a syndrome manifested by multiple cancers, and multiple cancers take years or even decades to occur gradually, so in the early stage of the disease, one or several cancers may appear, but it did not meet the diagnostic criteria for Li-Fraumeni syndrome, and Fraumeni syndrome may be diagnosed if it meets the diagnostic criteria in the future. Which means that these embryonal RMS patients may eventually be diagnosed with Li-Fraumeni syndrome. Other studies have also reached similar conclusions (3, 5, 7). Since Li-Fraumeni syndrome has a clear family history, the probability that high-risk family members will develop invasive malignant tumors before the age of 30 years is as high as 50%. Therefore, an understanding of the patients’ family history is important in order to establish which ones require more frequent and detailed screenings and helps determine the etiology of SMN. Time since diagnosis was found to be another risk factor for the development of SMN. Some studies have suggested that the main factors associated with the development of SMN were internal, such as cancer susceptibility (including a family history of Li-Fraumeni syndrome) rather than external such as radiotherapy and chemotherapy. Another important issue is whether the incidence of SMNs affects both solid tumors and hematological malignancies. It has been believed that the increase in the incidence of SMNs is significant in both types, yet the findings of our study indicate that the SIR for SMNs in all solid tumors was 1.95 (95% CI: 1.54 – 2.44, *p* < 0.05), but it was not statistically significant in hematological malignancies. In addition, there was an increased risk of some specific types of hematological malignancies, such as non-lymphocytic leukemia. Heyn et al. showed that the rates of solid SMNs (represented by osteosarcomas) and hematological malignancies (represented by acute non-lymphocytic leukemia) were significantly increased in RMS patients (3), whereas Archer et al. found that the increased risk of SMNs was only significant in solid tumors (16). This could be related to the sample size of the different studies, duration of the follow-up as well as the evolvement of systemic treatment.

This study has several limitations. Firstly, there are biases inherent to the retrospective design. Moreover, detailed treatment information, such as the exposure range of radiotherapy target areas, as well as the chemotherapy regimen and dose could not be acquired from the SEER database. Lastly, information on the tumor size, RMS remission/relapse and presence of RMS in addition to the sequence of radiotherapy and chemotherapy were not available.

## Conclusion

This study is a population cohort-based study that objectively describes the occurrence of SMN and its risk factors in patients with rhabdomyosarcoma. We found that the histologic subtypes and time since diagnosis were independent risk factors for the development of SMNs in patients with RMS. The results of this study can help with individualized screening approaches and follow-up timelines of RMS survivors. In the future, we will focus on the survival of RMS cohort and the occurrence of SMN through longer follow-up.

## Data Availability Statement

The raw data supporting the conclusions of this article will be made available by the authors, without undue reservation.

## Ethics Statement

Ethical review and approval was not required for the study on human participants in accordance with the local legislation and institutional requirements. Written informed consent from the participants’ legal guardian/next of kin was not required to participate in this study in accordance with the national legislation and the institutional requirements.

## Author Contributions

HZ and FZ conceptualized and designed the study. ZL and HG reviewed the literature. JS and JM collected data and performed statistical analysis. WW and ZM drafted the manuscript. All authors revised the manuscript. All authors contributed to the article and approved the submitted version.

## Funding

This work was supported by the National Key Research and Development Plan, the Ministry of Science and Technology of the People’s Republic of China [grant number 2016YFC0105207].

## Conflict of Interest

The authors declare that the research was conducted in the absence of any commercial or financial relationships that could be construed as a potential conflict of interest.

## Publisher’s Note

All claims expressed in this article are solely those of the authors and do not necessarily represent those of their affiliated organizations, or those of the publisher, the editors and the reviewers. Any product that may be evaluated in this article, or claim that may be made by its manufacturer, is not guaranteed or endorsed by the publisher.
